# Mammalian autophagy is essential for hepatic and renal ketogenesis during starvation

**DOI:** 10.1038/srep18944

**Published:** 2016-01-06

**Authors:** Ayano Takagi, Shinji Kume, Motoyuki Kondo, Jun Nakazawa, Masami Chin-Kanasaki, Hisazumi Araki, Shin-ichi Araki, Daisuke Koya, Masakazu Haneda, Tokuhiro Chano, Taiji Matsusaka, Kenji Nagao, Yusuke Adachi, Lawrence Chan, Hiroshi Maegawa, Takashi Uzu

**Affiliations:** 1 Department of Medicine, Shiga University of Medical Science, Tsukinowa-Cho, Seta, Otsu, Shiga 520-2192, Japan; 2 Division of Diabetology & Endocrinology, Kanazawa Medical University, Uchinada-machi, Kahoku-Gun, Ishikawa 920-0293, Japan; 3 Division of Metabolism and Biosystemic Science, Department of Internal Medicine, Asahikawa Medical University, Midorigaoka Higashinijyo, Asahikawa, Hokkaido 078-8510, Japan; 4 Department of Clinical Laboratory Medicine, Shiga University of Medical Science, Tsukinowa-Cho, Seta, Otsu, Shiga 520-2192, Japan; 5 Institute of Medical Science and Department of Internal Medicine, Tokai University School of Medicine, Bohseidai, Isehara, Kanagawa 259-1193, Japan; 6 Frontier Research Labs, Institute for Innovation, Ajinomoto Co., Inc., Suzuki-cho, Kawasaki, Kanagawa 210-8681, Japan; 7 Department of Medicine, Baylor College of Medicine, One Baylor Plaza, Houston, TX 77030, USA

## Abstract

Autophagy is an intracellular degradation system activated, across species, by starvation. Although accumulating evidence has shown that mammalian autophagy is involved in pathogenesis of several modern diseases, its physiological role to combat starvation has not been fully clarified. In this study, we analysed starvation-induced gluconeogenesis and ketogenesis in mouse strains lacking autophagy in liver, skeletal muscle or kidney. Autophagy-deficiency in any tissue had no effect on gluconeogenesis during starvation. Though skeletal muscle- and kidney-specific autophagy-deficiency did not alter starvation-induced increases in blood ketone levels, liver-specific autophagy-deficiency significantly attenuated this effect. Interestingly, renal as well as hepatic expression of HMG-CoA synthase 2 increased with prolonged starvation. Furthermore, during starvation, mice lacking autophagy both in liver and kidney showed even lower blood ketone levels and physical activity than mice lacking autophagy only in liver. Starvation induced massive lipid droplet formation in extra-adipose tissues including liver and kidney, which was essential for ketogenesis. Moreover, this process was impaired in the autophagy-deficient liver and kidney. These findings demonstrate that hepatic and renal autophagy are essential for starvation-induced lipid droplet formation and subsequent ketogenesis and, ultimately, for maintaining systemic energy homeostasis. Our findings provide novel biological insights into adaptive mechanisms to combat starvation in mammals.

All organisms have evolved tightly regulated mechanisms to maintain systemic energy homeostasis during life-threatening starvation. Autophagy is an evolutionarily conserved intracellular catabolic process that is activated in lower organisms during nutrient depletion^1^,^2^. Lower organisms with deficient autophagy cannot survive this potentially lethal situation^3^, indicating that autophagy is critical during starvation.

Accumulating evidence shows that autophagy is essential to protect tissues and cells against various types of cytotoxic stresses and that it regulates a wide range of physiological processes in mammals^1^,^2^. Autophagy is activated during prolonged starvation in mammalian tissues^4^. Systemic autophagy-deficient mice die immediately after birth^5^,^6^, suggesting that they cannot survive nutrient insufficiency during the neonatal period. Accordingly, autophagy would be expected to play an adaptive role in overcoming starvation across species. However, the physiological role of starvation-induced autophagy in mammals has not been fully elucidated.

Gluconeogenesis and ketogenesis are critical for maintaining energy homeostasis and physiological activity during starvation in mammals and are coordinately regulated in several tissues. The liver is essential for both gluconeogenesis and ketogenesis^7^,^8^ and the kidney also has a potential capacity for these processes^8^,^9^,^10^. Skeletal muscle provides glycogenic and ketogenic amino acids during starvation^11^ and fatty acids released from adipose tissue play critical roles in gluconeogenesis and ketogenesis^12^. Because starvation induces autophagy in all these organs, autophagy may be involved in regulating gluconeogenesis and/or ketogenesis in mammals^4^. Thus, this study aimed to determine tissue-specific roles of autophagy in gluconeogenesis and ketogenesis during starvation in mice with autophagy deficiency in selected tissues.

## Results

### Role of hepatic autophagy in gluconeogenesis and ketogenesis during starvation

**Figure 1:**
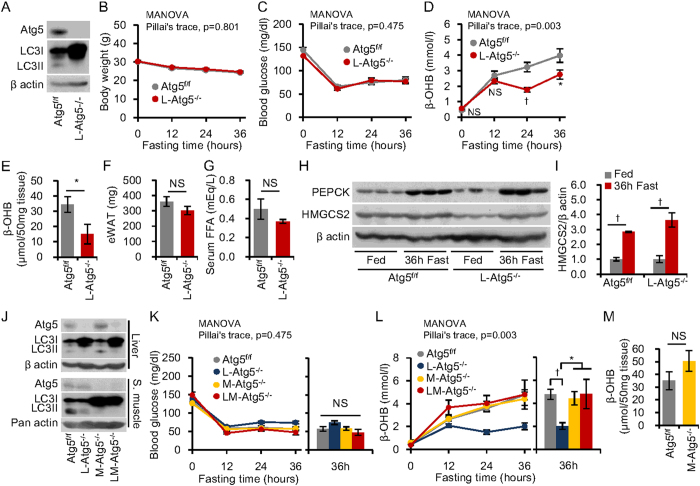
Starvation-induced gluconeogenesis and ketogenesis in liver- and muscle-specific Atg5^−/−^ mice. (**A**) Liver-specific autophagy-deficient mice (L- Atg5^−/−^) were generated by crossbreeding Atg5flox/flox (Atg5^f/f^) mice with albumin-Cre mice. Western blots for Atg5 and LC3II/LC3I proteins in liver tissues of mice fasted for 36 h. An absence of LC3II protein and increased LC3I protein indicate functional deletion of autophagy; β-actin was used as loading control. (**B–D**) Body weight (**B**), blood glucose (**C**), plasma β-hydroxybutyrate (β-OHB) levels (**D**) during 36 h fasting (n = 10 per group). (**E**) β-OHB concentrations in liver homogenates from Atg5^f/f^ mice and L- Atg5^−/−^ mice at 36 h fasting (n = 10 per group). (**F**) Weight of epigonadal white adipose tissue of Atg5^f/f^ mice and L- Atg5^−/−^ mice under ad libitum feeding. (**G**) Serum free fatty acid (FFA) levels in Atg5^f/f^ mice and L- Atg5^−/−^ mice after 36 h fasting (n = 5 per group). (**H**) Western blots for phosphoenolpyruvate carboxykinase (PEPCK) and HMG-CoA synthase 2 (HMGCS2) in liver tissue, with β-actin as loading control. (**I**) Quantitative analysis of the ratio of HMGCS2 to β-actin, with data represented as a fold-increase over levels in fed animals (n = 3 per group). (**J**) Skeletal muscle-specific autophagy-deficient mice (M-Atg5^−/−^) were generated by crossbreeding Atg5 ^f/f^ mice with Mlc-1f-Cre mice. L-Atg5^−/−^ mice were generated as described above and LM-Atg5^−/−^ are mice with the double knockout, in both liver and skeletal muscle. Western blots for Atg5 and LC3II/LC3I proteins in liver and skeletal muscle tissues after 36 h fasting indicated liver and skeletal muscle autophagy deficiency. β-actin and pan-actin were used as loading controls for the samples from liver and skeletal muscle, respectively. (**K,L**) Blood glucose (**K**) and β-OHB levels (**L**) during 36 h fasting (n = 12 for Atg5^f/f^, n = 10 for L-Atg5^−/−^, n = 5 for M-Atg5^−/−^, and n = 4 for LM-Atg5^−/−^). (**M**) β-OHB concentrations in skeletal muscle homogenates. Data are means ± SEM. *P < 0.05, ^†^P < 0.01, NS indicates not statistically significant.

Because the liver is principally involved in gluconeogenesis and ketogenesis during starvation^7^, we initially studied both processes in liver-specific autophagy-deficient mice. These mice were generated by crossbreeding Atg5^f/f^ mice with *albumin*-Cre mice. The protein encoded by autophagy-related gene *(Atg)* 5 is essential for autophagosome formation^5^. Western blotting confirmed that the livers from the mutant mice had deficient Atg5 protein, massive LC3I accumulation and an absence of LC3II, indicating deficient autophagy (Fig. 1A)^13^. We compared starvation-induced gluconeogenesis and ketogenesis in liver-specific *Atg5* knockout (L-Atg5^−/−^) and control (Atg5^f/f^) mice. After 36 h starvation, both L-Atg5^−/−^ and Atg5^f/f^ mice showed equivalent responses with respect to weight loss (Fig. 1B) and decreased blood glucose levels (Fig. 1C). In contrast, the starvation-induced increase in blood levels of β-hydroxybutyrate (β-OHB), a ketone body, was partially but significantly attenuated in L-Atg5^−/−^ mice as compared with control mice (Fig. 1D). Furthermore, L-Atg5^−/−^ mice had a significantly lower ketone content in the liver after 36 h fasting, as compared with Atg5^f/f^ mice (Fig. 1E).

Fatty acid released from adipose tissue during starvation is the precursor of ketone bodies^12^ and mitochondria-localised 3-hydroxy-3-methylglutaryl-coenzyme A synthase 2 (HMGCS2) is a rate-limiting enzyme of ketogenesis. Though levels of β-OHB during starvation were lower in L-Atg5^−/−^ mice (Fig. 1D), the epididymal fat volume in L-Atg5^−/−^ mice fed ad libitum (Fig. 1F) and plasma FFA levels after 36 h starvation (Fig. 1G) were the same as those in Atg5^f/f^ mice. Furthermore, starvation-induced increases in protein levels of HMGCS2 and phosphoenolpyruvate carboxykinase (PEPCK), a rate-limiting enzyme of gluconeogenesis, were not affected by the liver-specific autophagy deficiency (Fig. 1H,I).

These findings suggest that hepatic autophagy is involved in ketogenesis, but not in gluconeogenesis, during starvation. However, ketogenesis in L-Atg5^−/−^ mice was partially impaired and the reason for this, as well as the mechanisms underlying impaired ketogenesis mediated by an autophagy deficiency, remained unclear. To address the reason for the partial impairment, we postulated that organs other than the liver compensated for impaired hepatic ketone production in L-Atg5^−/−^ mice. This led us to examine mice with autophagy deficiencies in selected other tissues.

### Role of amino acid metabolism and skeletal muscle autophagy in gluconeogenesis and ketogenesis during starvation

Among several tissues associated with glucose and lipid metabolism during starvation, skeletal muscle is a key source of gluconeogenic and ketogenic amino acids^11^. We compared concentrations of ketogenic amino acids in the liver, skeletal muscle, and plasma of Atg5^f/f^ and L-Atg5^−/−^ mice after 36 h starvation to assess potential involvement of ketogenic amino acids in residual ketogenesis in the starved L-Atg5^−/−^ mice. Plasma levels of several ketogenic amino acids, such as leucine and isoleucine, significantly increased in L-Atg5^−/−^ mice, compared with in Atg5^f/f^ mice, under 36 h starvation (Table 1). Accordingly, the tissue contents of these ketogenic amino acids tended to increase in the livers of L-Atg5^−/−^ mice, though the difference from levels in Atg5^f/f^ mice were not statistically significant (Table 1).

We next generated skeletal muscle-specific *Atg5* knockout mice (M-Atg5^−/−^) using skeletal muscle-specific Cre (*Mlc-1f-Cre*) transgenic mice^14^ and *Atg5* double-knockout mice lacking the *Atg5* gene in liver and muscle tissues (LM-Atg5^−/−^) (Fig. 1J). These mice enabled us to examine effects of autophagy deficiency in skeletal muscle on gluconeogenesis and ketogenesis. Blood glucose and β-OHB levels did not significantly differ between starved M-Atg5^−/−^ and Atg5^f/f^ mice and β-OHB levels were not further reduced in LM-Atg5^−/−^, as compared with L-Atg5^−/−^ mice (Fig. 1K,L). Instead, the partially impaired ketogenesis in L-Atg5^−/−^ mice was restored in LM-Atg5^−/−^ mice (Fig. 1L). Moreover, there was no significant difference between M-Atg5^−/−^ and Atg5^f/f^ mice in skeletal muscle ketone content after 36 h fasting (Fig. 1M). These results indicated that skeletal muscle autophagy was not contributing to the residual ketone levels in the starved L-Atg5^−/−^ mice, though metabolic alteration of ketogenic amino acids might be involved in the mechanism compensating for impaired ketogenesis in L-Atg5^−/−^ mice.

### Role of renal autophagy in gluconeogenesis and ketogenesis during starvation

It is well known that the kidney regulates energy homeostasis during starvation via gluconeogenesis^8^. Interestingly, some reports have shown that the kidney has an ability to generate ketone bodies^8^,^9^,^10^ and that renal ketogenic activity, along with HMGCS2 expression, were enhanced under diabetic conditions^10^. Therefore, we examined the possibility that the kidney compensated for an impairment of ketogenesis in L-Atg5^−/−^ mice during starvation.

**Figure 2:**
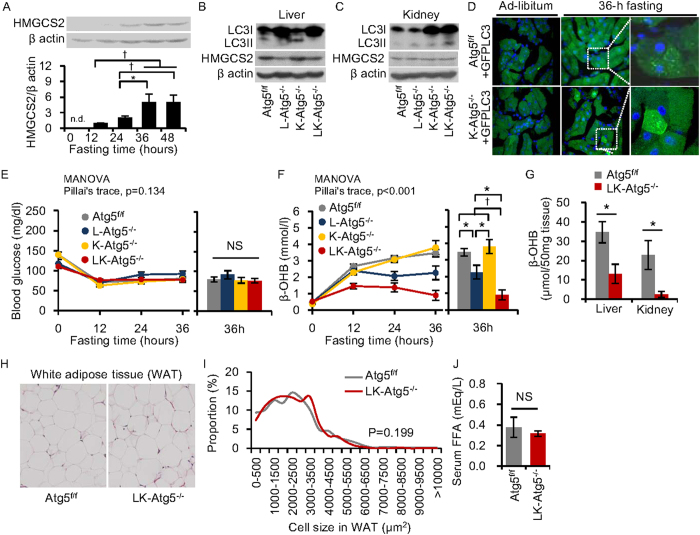
Starvation-induced gluconeogenesis and ketogenesis in liver- and kidney-specific Atg5^−/−^ mice. (**A**) Time-course analysis of HMG-CoA synthase 2 (HMGCS2) protein levels in kidney from Atg5^f/f^ mice by western blotting. (**B,C**) Kidney proximal tubular cell-specific autophagy-deficient mice (K-Atg5^−/−^) were generated by crossbreeding Atg5^f/f^ mice with Kap-Cre mice. L-Atg5^−/−^ mice were generated as described for Fig. 1 and LK-Atg5^−/−^ are mice with the double knockout, in both liver and kidney. Western blots for LC3II/LC3I proteins in liver and kidney tissues after 36 h fasting indicated liver and kidney autophagy deficiency. HMGCS2 expression levels of liver and kidney were also determined by western blots. (**D**) Kidney proximal tubular cells in K-Atg5^−/−^ + GFP-LC3 mice and Atg5^f/f^ + GFP-LC3 mice. Original magnification: 400×. (**E,F**) Blood glucose (**E**) and β-hydroxybutyrate (β-OHB) levels (**F**) during 36 h fasting (n = 21 for Atg5^f/f^, n = 9 for L-Atg5^−/−^, n = 8 for K-Atg5^−/−^, and n = 10 for LK-Atg5^−/−^). (**G**) β-OHB concentrations in liver and kidney homogenates from Atg5^f/f^ and LK-Atg5^−/−^ mice at 36 h fasting. (**H**) Representative pictures of haematoxylin and eosin stain of white adipose tissues in Atg5^f/f^ and LK-Atg5^−/−^ mice under ad-libitum feeding. Original magnification: 100×. (**I**) Proportion of cell size in white adipose tissue from Atg5^f/f^ and LK-Atg5^−/−^ mice under ad-libitum feeding. (**J**) Serum free fatty acid (FFA) levels in Atg5^f/f^ mice and LK- Atg5^−/−^ mice after 36 h fasting (n = 5, each). Data are means ± SEM. *P < 0.05, ^†^P < 0.01, NS indicates not statistically significant.

Initially, we evaluated levels of HMGCS2 expression in the mouse kidney during starvation. HMGCS2 protein was undetectable in kidneys from control mice fed ad libitum, but became detectable after 12 h starvation and increased further as starvation was prolonged (Fig. 2A). These findings suggested that renal ketogenesis is particularly important when starvation is prolonged.

We then generated kidney proximal tubular cell-specific *Atg5* knockout mice (K-Atg5^−/−^) from proximal tubular cell-specific Cre (*Kap-Cre*) mice^15^ and *Atg5* double-knockout mice lacking *Atg5* in the liver and kidney (LK-Atg5^−/−^). Deficient conversion of LC3I to LC3II was observed in the liver homogenates from L- and LK- Atg5^−/−^ mice (Fig. 2B). In addition to observing massive accumulation of LC3I protein in the total kidney homogenates from K- and LK-Atg5^−/−^ mice (Fig. 2C), we confirmed that there was proximal tubular cell-specific autophagy deficiency by crossbreeding K-Atg5^−/−^ with GFP-LC3 mice, a strain generated to monitor autophagy in mouse tissues^4^ (Fig. 2D). In the proximal tubular cells of Atg5^f/f^ mice crossbred with GFP-LC3 transgenic mice, substantial dotted fluorescence from GFP-LC3, indicating active autophagosome formation, was observed after 36 h fasting (Fig. 2D). However, this fasting-induced formation of GFP-LC3 dots was impaired, and GFP signals instead became widely distributed in the cytoplasm, in proximal tubular cells of K-Atg5^−/−^ mice crossbred with GFP-LC3 transgenic mice, even after 36 h fasting (Fig. 2D).

We monitored blood glucose and β-OHB levels in LK-Atg5^−/−^ mice during 36 h starvation. Changes in blood glucose levels did not significantly differ among the four mouse strains (Fig. 2E) and changes in β-OHB levels in K-Atg5^−/−^ mice were equivalent to those in control mice (Fig. 2F). In addition, control Atg5^f/f^, L-Atg5^−/−^ and K-Atg5^−/−^ mice showed normal physical activity even after 36 h fasting (Supplemental movies 1, 2, 3). However, the starvation-induced increase in blood β-OHB was further attenuated in LK-Atg5^−/−^, as compared with in L-Atg5^−/−^, mice (Fig. 2F), though hepatic and renal HMG-CoA synthase levels were not affected by either autophagy deficiency (Fig. 2B,C). Furthermore, LK-Atg5^−/−^ mice showed significantly lower ketone contents in the liver and kidney after 36 h fasting (Fig. 2G). As a consequence, the impairment of ketogenesis resulted in low physical activity in LK- Atg5^−/−^ mice after 36 h starvation (Supplemental movie 4).

To exclude the possibility that LK- Atg5^−/−^ mice showed an impairment of adipogenesis, which might result in deficiencies of FFA supply and subsequent ketogenesis, we analysed adipocyte size in epigonadal white adipose tissue. There was no difference between LK-Atg5^−/−^ mice and Atg5^f/f^ mice in adipocyte size of epigonadal white adipose tissues under ad-libitum feeding (Fig. 2H,I) and the two strains also had the same starvation-induced increase in serum FFA levels (Fig. 2J). These results suggest that hepatic and renal autophagy regulates starvation-induced ketogenesis without affecting adipogenesis.

### Role of autophagy in starvation-induced lipid droplet formation in tissues

Deficient autophagy resulted in impaired ketogenesis but did not affect HMGCS expression, adipogenesis and starvation-induced FFA release. To clarify the mechanisms underlying this impaired ketogenesis, we examined whether deficient autophagy affected intracellular fatty acid metabolism, a source of ketone bodies. Fatty acids released from adipose tissues are reabsorbed into peripheral tissues from the bloodstream, temporarily accumulating as lipid droplets until they are utilised for β-oxidation^16^. Because autophagy was reported to be associated with lipid droplet formation and/or lipolysis^17^,^18^,^19^, we investigated which of these processes was affected by deficient autophagy during starvation.

**Figure 3:**
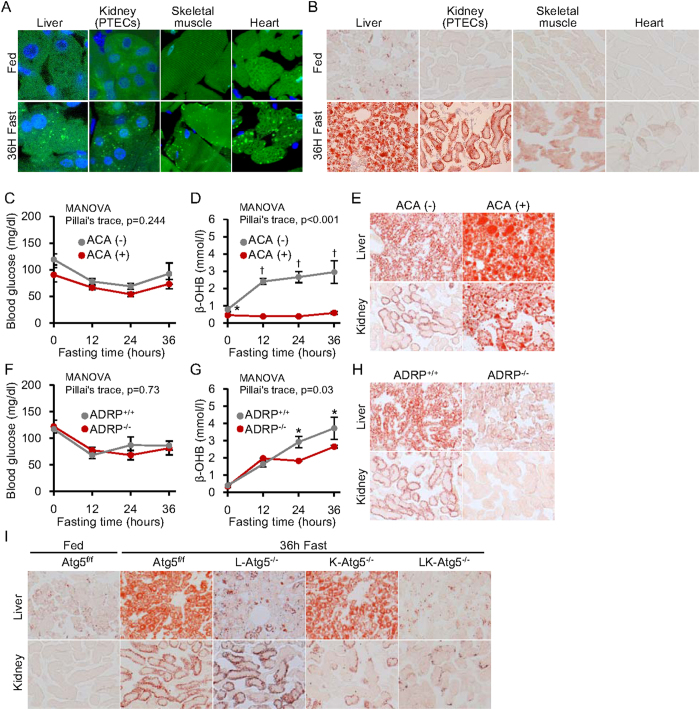
Effect of autophagy-deficiency on starvation-induced lipid droplet formation in liver and kidney. (**A**) Starvation-induced autophagy activation was detected in liver, skeletal muscle, heart and kidney proximal tubules of GFP-LC3 transgenic mice as green dotted fluorescence signals. Original magnification: 1000×. (**B**) Starvation-induced triglyceride accumulation in various tissues of mice, determined by Oil Red O staining. Original magnification: 100×. (**C–E**) Animal study using L-aminocarnitine (L-ACA) treatment to impair fatty acid utilization in the mitochondria. (**C,D**) Blood glucose (**C**) and β-hydroxybutyrate (β-OHB) levels (**D**) in mice, with and without L-ACA treatment, during 36 h fasting (n = 9 per group). (**E**) Oil Red O staining to visualise lipid droplets in liver and kidney from mice, with and without L-ACA treatment, after 36 h starvation. Original magnification: 100×. (**F–H**) Animal study comparing adipose differentiation-related protein knockout (ADRP^−/−^) to wild type (ADRP^ + / + ^) mice. ADRP is a protein essential to the formation of lipid droplets. (**F,G**) Blood glucose (**F**) and β-OHB levels (**G**) in ADRP^−/−^ and ADRP^ + / + ^mice during 36 h fasting (n = 4 per group). (**H**) Oil Red O stain showing lipid droplets in liver and kidney tissues after 36 h starvation. Original magnification: 100×. (**I**) Oil Red O stain showing lipid droplets in the autophagy-deficient tissues of L-Atg5^−/−^, K-Atg5^−/−^ and LK-Atg5^−/−^ mice after 36 h starvation. Original magnification: 100×. Data are means ± SEM. *P < 0.05, ^†^P < 0.01.

Activation of starvation-induced autophagy in the liver, heart, skeletal muscle, and kidney proximal tubular cells was confirmed in GFP-LC3 transgenic mice (Fig. 3A). Interestingly, in all examined tissues, starvation-induced lipid droplet formation, visualized with Oil Red O staining, was detected (Fig. 3B). To examine the roles of β-oxidation and starvation-induced lipid accumulation in ketogenesis, we treated mice with L-aminocarnitine (L-ACA), an inhibitor of mitochondrial long-chain fatty acid β-oxidation^20^. We also used adipose differentiation-related protein (ADRP)-knockout mice, which exhibit impaired lipid droplet formation^21^.

Glucose metabolism was not affected during starvation in both L-ACA-treated and ADRP-knockout mice (Fig. 3C,F), similar to the observations in autophagy-deficient mice. Treatment of wild type mice with L-ACA impaired starvation-induced lipolysis, resulting in impaired ketogenesis accompanied by the accumulation of large lipid droplets in the liver and kidney (Fig. 3D,E). Furthermore, mice with a systemic ADRP-deficiency had defective lipid droplet formation in the liver and kidney and impaired ketogenesis during starvation (Fig. 3G,H). These results suggest that lipid droplet formation in tissues during fasting is essential for ketogenesis.

We then stained the livers and kidneys of tissue-specific Atg5-deficient mice with Oil Red O. Lipid droplet formation was significantly reduced in the autophagy-deficient liver and kidney proximal tubular cells (Fig. 3I), suggesting that Atg5-regulated autophagy is critical for starvation-induced lipid droplet formation.

## Discussion

The most important finding of this study is that autophagy is essential for starvation-induced lipid droplet formation and subsequent ketogenesis in adult mice. Many reports have revealed that cytotoxic stress-induced autophagy plays a cytoprotective role and its alteration is involved in the pathogenesis of many modern diseases^1^,^2^. However, until recently, the physiological role of autophagy in adult mammalian survival during starvation has not been well addressed. Our results provide novel insights into the physiology of autophagy in mammals.

Both gluconeogenesis and ketogenesis are critical for survival in starving adult mammals. We concluded from our findings in tissue-specific Atg5-deficient mice that Atg5-related autophagy is essential for ketogenesis, rather than gluconeogenesis, in mammals. In a previous report, liver-specific Atg7-deficient mice become hypoglycaemic after 24 h starvation^22^. This observation was not consistent with our results using Atg5-deficient mice. Further research is needed to elucidate the differential roles of *Atg* genes in gluconeogenesis and ketogenesis in mammals.

Two theories have been proposed regarding how autophagy influences lipid metabolism^17^,^18^,^19^,^23^. One is activation of lysosomal lipolysis from lipid droplets, known as lipophagy^19^, and the other is promotion of lipid droplet formation^17^. We found impaired starvation-induced lipid droplet formation in Atg5-deficient liver and kidney, in agreement with reports that *Atg* genes are involved in formation of lipid droplets in both *Caenorhabditis elegans* and cultured mammalian cells^17^,^23^,^24^. However, it remains possible that autophagy can regulate both lipid droplet formation and lipolysis during starvation because autophagy disrupts a step in lipid droplet formation that is upstream in the cellular process of lipolysis.

Though starvation-induced lipid droplet formation in extra-adipose tissues was previously reported^16^, its physiological role has long been unknown. During starvation, glucose utilization is suppressed in extra-adipose tissues and, instead, fatty acids are used as a fuel to maintain whole-body energy homeostasis. Our observation that disrupted lipid droplet formation in extra-adipose tissues resulted in impaired ketogenesis suggests that lipid droplet formation in extra-adipose tissues originally evolved as a critical system for overcoming starvation in times of scarcity; however ectopic lipid accumulation in liver and kidney is now considered a pathogenic change in obesity and diabetes. We further found that starvation-induced lipid droplets were much more abundant in the liver and kidney than in other tissues such as skeletal muscle and heart, suggesting that the supply of abundant fatty acids to these two organs for generating ketone bodies is of the highest priority for mammalian survival during starvation.

Kuma *et al*. reported that amino acid concentrations were lower in plasma and tissues of systemic Atg5-deficient neonatal mice, a possible explanation for their early postnatal death^5^. Because levels of blood glucose, ketones and FFAs in normal neonatal mice are commonly very low even under starvation, amino acids might provide a unique energy source for neonatal mice during the postnatal period. In contrast, in adult mice, gluconeogenesis and ketogenesis are important for overcoming periods of starvation. Based on the results of our study, autophagy in adult mammals plays a critical role in starvation-induced ketogenesis via regulation of tissue lipid metabolism. Taken together, these findings suggest the interesting perspective that autophagy plays distinct roles in neonatal and in adult animals relative to their adaptive mechanisms against starvation. Starvation-induced autophagy may be critical to amino acid metabolism and lipid metabolism during starvation in neonate and adult mammals, respectively.

In addition to providing information about the role of autophagy in ketogenesis, our findings contribute insights into the physiology of the mammalian kidney during starvation. Kidney proximal tubular cells produce not only glucose but also ketone bodies under starvation. One report, published in the 1990s, showed that renal ketogenesis is enhanced during hepatic inflow occlusion^9^. Another report showed that renal ketogenic activity was increased, along with HMGCS2 expression, under diabetic conditions^10^. These studies demonstrated that the kidney can generate ketone bodies under some conditions. However, the detailed mechanisms of renal ketogenesis and its physiological importance have remained unknown. In our study, both HMGCS2 expression and lipid accumulation were increased during starvation, even in the kidneys of normal mice. This suggests that the kidney, along with the liver, produces ketone bodies even when hepatic ketogenesis is functioning normally. While K-Atg5^−/−^ mice showed normal ketone levels during starvation, LK-Atg5^−/−^ mice had almost completely impaired ketogenesis. These results suggest that the normal liver can fully compensate for a disturbance in renal ketogenesis because ketogenic capacity in the liver is very high. They further suggest that the kidney plays a supportive role in starvation-induced ketogenesis, similar to its role in gluconeogenesis.

Unlike LK-Atg5^−/−^ mice, LM-Atg5^−/−^ mice did not show further decreases in β-OHB during starvation, suggesting that autophagy in skeletal muscle is not involved in the mechanism of compensation for impaired ketogenesis in L-Atg5^−/−^ mice. Instead, the partially impaired ketogenesis in L-Atg5^−/−^ mice was, surprisingly, restored in LM-Atg5^−/−^ mice. This result raises the possibility that autophagy in skeletal muscle plays an opposite role in ketogenesis to those of hepatic and renal autophagy or that it provides a positive regulatory mechanism for ketone body consumption in skeletal muscle. However, our study did not distinguish between these possibilities and further investigation will be needed to clarify the exact role of autophagy in the skeletal muscle during starvation.

One remaining question is why residual very low levels of plasma ketone were still observed in the LK-Atg5^−/−^ mice and we speculate on potential reasons. First, since because kinds of ketogenic amino acids were increased in plasma and liver of L-Atg5^−/−^ mice under starvation, the metabolic alteration of ketogenic amino acids might contribute to residual ketogenesis in LK-Atg5^−/−^ mice. Another possibility is that other organs or other cells within the kidney might generate ketone bodies during starvation in LK-Atg5^−/−^ mice. Indeed, strong HMGCS expression was previously reported in renal glomeruli^10^, suggesting that these cells, in addition to proximal tubular cells, might contribute to renal ketogenesis. This would explain the normal ketone levels observed in the starved mice with proximal tubular cell specific Atg5 deletion. In addition, it has been reported that intestinal cells have ketogenic genes^25^,^26^, suggesting they can produce ketone bodies. Further experiments examining these possibilities could provide additional insights into the physiology of starvation.

In conclusion, starvation-induced autophagy in the liver plays an essential role in ketogenesis to maintain energy homeostasis, and under starvation, the kidney plays a supporting role in ketogenesis. In addition, autophagy-regulated lipid droplet formation in the liver and kidney is required for ketogenesis. These results provide important insights into mammalian autophagy and renal physiology during starvation.

## Methods

### Animals

All animal handling and experimentation was conducted according to the guidelines of the Research Center for Animal Life Science at Shiga University of Medical Science. All experimental protocols were approved by the Gene Recombination Experiment Safety Committee and the Research Center for Animal Life Science at Shiga University of Medical Science. Tissue-specific autophagy-deficient mouse models were generated using the Cre-loxP system. We used Atg5-floxed (Atg5^f/f^) mice^27^, transgenic Albumin-Cre mice (Jackson Laboratory, Bar Harbor, ME, USA), KAP-Cre^15^, or MLC1f-Cre^14^. PCR was performed to detect the Atg5^flox^ alleles or Cre transgenes, using the following primers. For wild-type Atg5 and Atg5^flox^ alleles, exon3-1, 5′-GAATATGAAGGCACACCCCTGAAATG-3′; short2, 5′-GTACTGCATAATGGTTTAACTCTTGC-3′; check2, 5′-ACAACGTCGAGCACAGCTGCGCAAGG-3′; 5L2, 5′-CAGGGAATGGTGTCTCCCAC-3′; cre1, 5′-AGGTTCGTTCACTCATGGA-3′; cre2, 5′-TCGACCAGTTTAGTTACCC-3′^27^.

To identify LK-Atg5^−/−^ mice, we used primers for Albumin-Cre (5′-AGGACATGGACAAGGTCGAG-3′ and 5′-TGGAGTGGCAACTTCCAAG-3′) and that for KAP-Cre (5′-GTCCATGGTGATACAAGGGACATC-3′ and 5′-CATAAAGGTCCTTCCCAAACCCCT-3′) together^15^. For LM-Atg5^−/−^ mice, we recognised autophagy deficiency in liver or skeletal muscle by western blotting for Atg5 protein using tissue homogenates collected from mice after starvation, analysis and sacrifice, because a MLC1fCre-specific PCR primer set was not available for this study.

Where indicated, C57BL6 mice were subcutaneously injected twice with L-ACA (30 mg/kg) or a phosphate-buffered saline (PBS) vehicle control at the beginning of the experiment and after 24 h starvation. At 12 wk of age, C57BL6 mice, ADRP knockout mice^21^ and mice with tissue-specific autophagy deficiencies were starved for 36 h beginning at 9 pm. For analysis of blood glucose and plasma ketone levels, blood was collected by tail vein sampling every 12 h during starvation. Following starvation and other treatment protocols, mice were sacrificed by pentobarbital and plasma and tissues collected for analysis.

### Blood glucose and ketone analysis

Blood glucose levels were measured using a Glutest sensor (Sanwa Kagaku, Nagoya, Japan). Plasma ketone levels were measured using a Precision Xceed (Abbott, Chiba, Japan). This handheld β-hydroxybutyrate test strip is highly sensitive and specific and gave results in good agreement with independent β-hydroxybutyrate assays conducted in the laboratory^28^.

### Tissue ketone analysis

We measured the levels of tissue ketone bodies with a β-hydroxybutyrate fluorometric assay kit (Cayman Chemical Co, Ann Arbor, MI, USA) according to the manufacturer’s instructions. Prior to dissection, each tissue was rinsed with a PBS solution to remove any red blood cells and clots. We used 50 mg tissue homogenate for each assay.

### Histological analysis

Tissue samples were frozen in OCT compound (Sakura, Torrance, CA, USA), cut into 5 μm sections, fixed in 10% formalin/0.05% picric acid cacodylate-buffer (0.1 M, pH 7.4) for 30 min and rinsed in PBS before staining with Oil Red O^29^. Cell size and cell counting analyses of adipocytes was performed with Image-Pro plus 7.0 (Media Cybernetics, Bethesda, MD, USA) after haematoxylin and eosin (HE) staining.

### Western blotting

Western blotting was performed as previously described^29^. Antibodies against Atg5 (Cell Signaling Technology; Beverly, MA, USA), LC3 (Novus Biologicals; Littleton, CO, USA), β-actin (Sigma Aldrich Co, St. Louis, MO, USA), Pan-actin (Santa Cruz Biotechnology, Santa Cruz, CA, USA), and HMGCS (Sigma Aldrich Co) were used for detecting the indicated proteins.

### Measurement of free fatty acid (FFA) levels

Plasma FFA levels were determined using a non-esterified fatty acid C-test kit (Wako Chemical Industries, Ltd., Osaka, Japan).

### Monitoring autophagy with GFP-LC3 transgenic mice

Starvation-induced activation of autophagy was detected in the liver, skeletal muscle, heart and kidney proximal tubules of GFP-LC3 transgenic mice as green dotted fluorescence signals. To induce autophagy, GFP-LC3 transgenic mice were starved for 36 h. The mice were euthanised, frozen tissue sections were prepared and autophagosomes were observed by confocal laser microscopy (LSM 510; Zeiss, Thornwood, NY, USA)^4^. To confirm proximal tubular cells-specific autophagy deficiency, GFP-LC3 mice were crossed with Atg5-floxed mice carrying the KAP-Cre gene to breed K-Atg5^−/−^ + GFP-LC3 mice.

### Measuring glucogenic and ketogenic amino acid levels

Liver and skeletal muscle samples were rinsed in PBS and homogenised. Tissue samples were deproteinised with 10% trichloroacetic acid and filtered. Plasma samples were mixed with 2 volumes 5% trichloroacetic acid, centrifuged (4 °C, 15 min, 10,000×g) and supernatants were filtered through a Microcon Ultracel YM-10 (Nihon Millipore, Tokyo, Japan). Amino acid concentrations in filtrates from all samples were measured with an automatic amino acid analyser (L-8800A; Hitachi, Tokyo, Japan)^30^.

### Statistical analysis

Results are expressed as means ± SEM. MANOVA followed by Tukey’s HSD test or Student’s t-test was used for time-course analyses. Student’s t test was used to assess differences between two groups. Cumulative survival was estimated with the Kaplan–Meier method. A *P* value <0.05 was considered statistically significant.

## Additional Information

**How to cite this article**: Takagi, A. *et al*. Mammalian autophagy is essential for hepatic and renal ketogenesis during starvation. *Sci. Rep*. **6**, 18944; doi: 10.1038/srep18944 (2016).

## Supplementary Material

Supplementary Movie S1

Supplementary Movie S2

Supplementary Movie S3

Supplementary Movie S4

Supplementary Movie Legends

## Figures and Tables

**Table 1:** Glucogenic and ketogenic amino acids levels in liver, skeletal muscle and plasma samples from Atg5-flox (Atg^f/f^) and liver-specific Atg5-deficient (L-Atg5^−/−^) mice after 36 h starvation.

	Atg5^f/f^ (n = 7)	L-Atg5^−/−^ (n = 7)	Student’s t test
Liver (pmol/mg)			
Leucine	226.1 ± 37.2	351.8 ± 79.0	n.s.
Lysine	541.3 ± 195.2	470.2 ± 134.1	n.s.
Isoleucine	100.7 ± 15.6	190.8 ± 60.0	n.s.
Phenylalanine	58.9 ± 5.0	81.1 ± 27.6	n.s.
Tyrosine	66.5 ± 9.8	82.5 ± 28.9	n.s.
Tryptophan	17.7 ± 1.9	20.7 ± 4.0	n.s.
Skeletal muscle (pmol/mg)			
Leucine	250.4 ± 43.4	312.3 ± 40.5	n.s.
Lysine	470.8 ± 73.3	460.7 ± 117.5	n.s.
Isoleucine	141.4 ± 14.4	151.4 ± 19.1	n.s.
Phenylalanine	108.4 ± 10.7	128.0 ± 20.8	n.s.
Tyrosine	105.5 ± 12.3	138.4 ± 40.0	n.s.
Tryptophan	27.5 ± 1.5	31.9 ± 4.6	n.s.
Plasma (μM)			
Leucine	214.1 ± 37.0	339.4 ± 35.7	P < 0.05
Lysine	240.0 ± 46.2	179.3 ± 18.6	n.s.
Isoleucine	109.7 ± 12.2	145.5 ± 10.6	P < 0.05
Phenylalanine	81.1 ± 4.9	91.4 ± 8.6	n.s.
Tyrosine	63.6 ± 9.1	69.6 ± 9.7	n.s.
Tryptophan	66.3 ± 4.9	77.4 ± 4.4	n.s.

n.s. indicates not statistically significant.
