# Supplementary material for: Mammalian autophagy is essential for hepatic and renal ketogenesis during starvation
Source: Sci Rep. 2016 Jan 6;6:18944. doi: 10.1038/srep18944 (PMC4702170; doi:10.1038/srep18944)
Supplement: Supplementary Movie Legends [file srep18944-s5.doc]

**Mammalian autophagy is essential for hepatic and renal ketogenesis during starvation**

Ayano Takagi,^1^ Shinji Kume,^1^ Motoyuki Kondo,^1^ Jun Nakazawa,^1^ Masami Chin-Kanasaki,^1^ Hisazumi Araki,^1^ Shin-ichi Araki,^1^ Daisuke Koya,^2^ Masakazu Haneda,^3^ Tokuhiro Chano,^4^ Taiji Matsusaka,^5^ Kenji Nagao,^6^ Yusuke Adachi,^6^ Lawrence Chan,^7^ Hiroshi Maegawa,^1^ Takashi Uzu^1^

^1^Department of Medicine, Shiga University of Medical Science, Tsukinowa-Cho, Seta, Otsu, Shiga 520-2192, Japan

^2^Division of Diabetology & Endocrinology, Kanazawa Medical University, Uchinada-machi, Kahoku-Gun, Ishikawa 920-0293, Japan

^3^Division of Metabolism and Biosystemic Science, Department of Internal Medicine, Asahikawa Medical University, Midorigaoka Higashinijyo, Asahikawa, Hokkaido 078-8510, Japan.

^4^Department of Clinical Laboratory Medicine, Shiga University of Medical Science, Tsukinowa-Cho, Seta, Otsu, Shiga 520-2192, Japan

^5^Institute of Medical Science and Department of Internal Medicine, Tokai University School of Medicine, Bohseidai, Isehara, Kanagawa 259-1193, Japan

^6^Frontier Research Labs, Institute for Innovation, Ajinomoto Co., Inc., Suzuki-cho, Kawasaki, Kanagawa 210-8681, Japan

^7^Department of Medicine, Baylor College of Medicine, One Baylor Plaza, Houston, TX 77030, USA.

**Supplemental Movie Legends**

**Supplemental Movie 1.** Physical activity of Control Atg5^f/f^ mice after 36-h starvation. Control Atg5^f/f^ mice showed normal physical activity even after 36-h starvation.

**Supplemental Movie 2.** Physical activity of liver-specific Atg5-deficient (L-Atg5^-/-^) mice after 36-h starvation. L-Atg5^-/-^ mice showed normal physical activity even after 36-h starvation.

**Supplemental Movie 3.** Physical activity of kidney proximal tubular cell-specific Atg5-deficient (K-Atg5^-/-^) mice after 36-h starvation. K-Atg5^-/-^ mice showed normal physical activity even after 36-h starvation.

**Supplemental Movie 4.** Physical activity of liver and kidney proximal tubular cell-specific Atg5-deficient (LK-Atg5^-/-^) mice after 36-h starvation. LK-Atg5-/- mice showed apparently lower physical activity after 36-h starvation compared with the other groups of mice (supplemental movie 1-3).
